# Evaluation of the Surface Topography of Microfinishing Abrasive Films in Relation to Their Machining Capability of Nimonic 80A Superalloy

**DOI:** 10.3390/ma17102430

**Published:** 2024-05-18

**Authors:** Katarzyna Tandecka, Wojciech Kacalak, Filip Szafraniec, Michał Wieczorowski, Thomas G. Mathia

**Affiliations:** 1 Department of Engineering and Informatics Systems, Faculty of Mechanical Engineering and Energy, Koszalin University of Technology, 75-620 Koszalin, Poland; wojciech.kacalak@tu.koszalin.pl (W.K.); filip.szafraniec@tu.koszalin.pl (F.S.); 2 Faculty of Mechanical Engineering, Institute of Applied Mechanics, Poznan University of Technology, 3 Piotrowo St., 60-965 Poznan, Poland; michal.wieczorowski@put.poznan.pl; 3 Laboratoire de Tribologie et Dynamique des Systemes (LTDS), Ecole Centrale de Lyon, Centre National de la Recherche Scientifique, 69134 Lyon, France; thomas.mathia@ec-lyon.fr

**Keywords:** surface finishing, abrasive film, finishing, abrasion, superfinishing, superalloy, machining capability, Nimonic 80A, Voronoi cells, nickiel material

## Abstract

This study investigates the surface topography of microfinishing abrasive films and their machining capability on the Nimonic 80A superalloy, a high-performance nickel-based alloy commonly used in aerospace and gas turbine engine applications. Surface analysis was conducted on three abrasive films with nominal grain sizes of 30, 15, and 9 μm, exploring wear patterns, contact frequency, and distribution. To assess the distribution of grain apexes, Voronoi cells were employed. Results revealed distinct wear mechanisms, including torn abrasive grains and cracked bond surfaces, highlighting the importance of efficient chip removal mechanisms in microfinishing processes. Larger grain sizes exhibited fewer contacts with the workpiece but provided more storage space for machining products, while smaller grain sizes facilitated smoother surface finishes. The research demonstrated the effectiveness of microfinishing abrasive films in reducing surface irregularities. Additionally, surface analysis of worn abrasive tools provided insights into wear mechanisms and chip formation, with the segmentation of microchips contributing to efficient chip removal. These findings underscore the significance of selecting appropriate abrasive films and implementing effective chip removal mechanisms to optimize microfinishing processes and improve surface finishing quality in advanced material machining applications. It is worth emphasizing that no prior research has investigated the microfinishing of components crafted from Nimonic 80A utilizing abrasive films, rendering this study truly unique in its contribution to the field.

## 1. Introduction

Nimonic 80A is a nickel-based superalloy renowned for its exceptional high-temperature strength [1,2], oxidation resistance [3], and creep resistance, making it a prime choice for demanding engineering applications [4], particularly in the aerospace and gas turbine industries [5]. Nimonic 80A alloy typically exhibits a yield strength ranging from 550 to 690 MPa, along with an ultimate tensile strength between 750 and 950 MPa. Its elongation at break typically falls within the range of 20% to 30%. Moreover, Nimonic 80A boasts an impressive creep rupture strength at elevated temperatures, with values surpassing 300 MPa at 650 °C. Additionally, its oxidation resistance is notable, with a protective oxide layer forming on its surface, allowing it to withstand temperatures exceeding 700 °C in oxidizing environments. This alloy derives its superior mechanical properties from a balanced composition of nickel, chromium, and titanium, along with traces of aluminum and carbon [6,7]. Such a composition provides excellent resistance to corrosion [8,9], thermal stability, and mechanical strength, making Nimonic 80A an ideal material for components subjected to extreme operating conditions [10]. In the aerospace sector, Nimonic 80A finds extensive use in manufacturing various rotating components, including turbine blades, turbine discs, and compressor rings, owing to its ability to withstand high temperatures and mechanical stress. Additionally, in gas turbine engines, Nimonic 80A is utilized for manufacturing critical parts like combustion chamber liners, exhaust valves, and nozzle guide vanes, where resistance to oxidation and creep deformation is paramount. The application of microfinishing with abrasive films in machining Nimonic 80A components extends beyond aerospace to various industrial sectors, including power generation [11], automotive [12,13], and marine propulsion. In power generation, Nimonic 80A-based turbine components require precise machining for optimal performance and efficiency, driving the adoption of microfinishing techniques. Similarly, in automotive and marine propulsion systems, where high-temperature and high-stress environments are prevalent, Nimonic 80A components undergo microfinishing to enhance durability and reliability. In summary, Nimonic 80A stands out as a high-performance superalloy renowned for its exceptional mechanical properties, making it a preferred choice for manufacturing rotating components in aerospace, gas turbine, and other high-temperature applications. Microfinishing with abrasive films emerges as a crucial machining process for achieving precise surface characteristics and dimensional accuracy in Nimonic 80A components across various industrial sectors. It is important to highlight that no research studies on microfinishing components made from Nimonic 80A using abrasive films were found. Therefore, this study should be considered unique [14].

**Figure 1:**
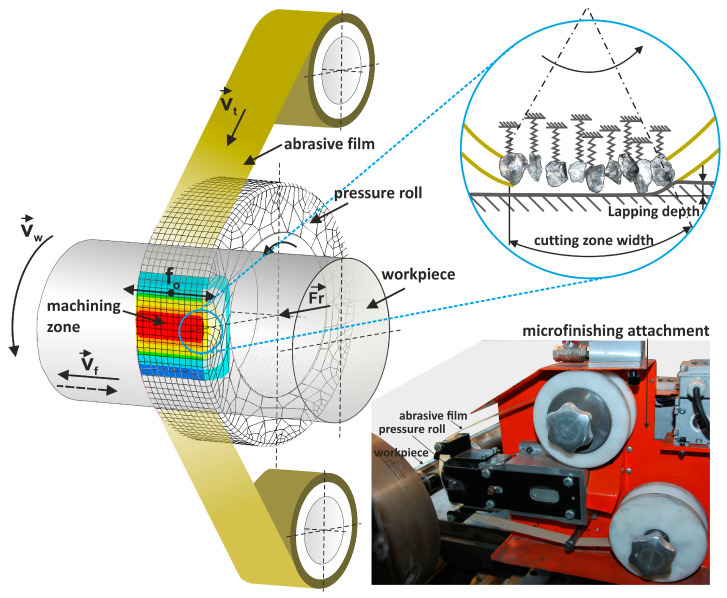
Kinematic diagram of rotary surface finishing using lapping films, where the following quantities are indicated on the diagram: *v_t_*—tool speed, *v_w_*—workpiece speed, *v_f_*—tool feed speed, *f_o—_*tool oscillation frequency, and *F_r_*—the pressure force of the pressing roller [40] and photograph of the research setup.

**Figure 2:**
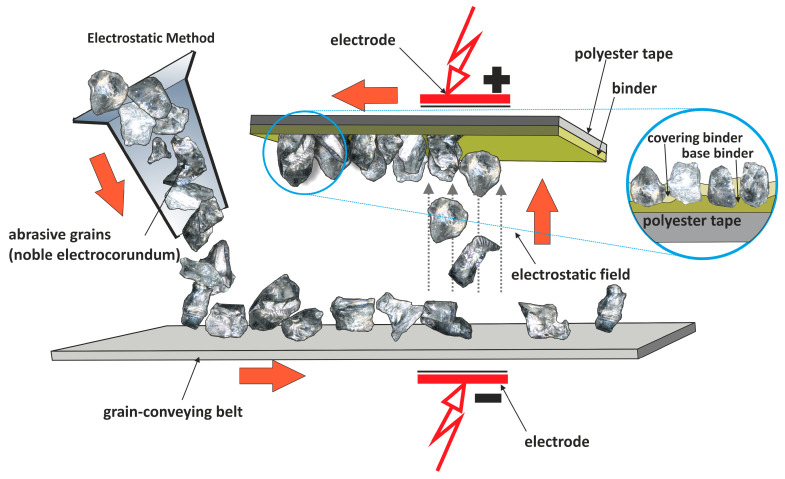
The production scheme in the electrostatic field of microfinishing films [41].

In the realm of microabrasive machining, exemplified by precision grinding and microfinishing, the depth of the tool’s intrusion into the workpiece material remains markedly shallower than the curvature of its edges, akin to the minute elevations present within the microcutting zone [15,16,17]. The inherent variability in the penetration of abrasive grains into the workpiece material stands as an undesirable yet inescapable trait of microcutting [18,19]. A contemporary approach to refining technical surfaces through microfinishing, employing abrasive films, has emerged as a paramount technique for refining external cylindrical and conical surfaces alongside flat end faces [20,21]. This method facilitates the efficient treatment of an array of materials, spanning metals, plastics, composite materials, ceramics, and super-hard materials [22,23,24]. Operating with precision abrasive films featuring a carrier constructed from delicate polyester film layered with one or more strata of abrasive grains [25,26], this process embodies a distinctive paradigm where the tool undergoes singular employment [27,28]. Initiating the microfinishing process entails unfurling the abrasive film from a spool and applying it against the workpiece with a force exerted by a pressure roller (Figure 1). Upon completion of its use, the spent film is wound back onto a roller, which concurrently functions as a driving mechanism. This disposability aspect sets this method apart from procedures reliant on infinite abrasive belts [29,30,31,32]. Tailored for microfinishing applications, the composition of microfinishing films is meticulously engineered, with abrasive grains embedded within a polymeric matrix through the utilization of electrostatic fields (Figure 2). This meticulous process yields sharper cutting edges, culminating in precise surface finishes. Widely embraced across sectors such as automotive, aerospace, and medical industries, these films epitomize excellence in achieving exacting surface quality. It is worth noting that in this type of machining, the workpiece rotates significantly faster relative to the tool, contributing to a faster and more efficient surface finishing process [33,34,35]. Additionally, the tool is used only once, meaning that specific abrasive grains are used only once as well. This allows for the preservation of their sharpness throughout the entire process, resulting in uniform and high-quality surface finishes [36,37,38]. Microfinishing, a critical process in surface engineering, plays a pivotal role in achieving precise surface characteristics essential for various industrial applications [39].

The present study delves into the intricate interplay between abrasive grain size, contact frequency, wear mechanisms, and chip removal efficiency in microfinishing operations. By understanding these dynamics, manufacturers can refine their processes to optimize surface quality and productivity. Surface finish quality is a paramount concern across industries ranging from aerospace to automotive, where even minor imperfections can compromise performance and durability. The selection of abrasive grain size profoundly influences surface finish, with finer grains typically associated with smoother surfaces.

However, the precise relationship between abrasive grain size and surface quality remains a subject of investigation. Existing research has explored the impact of abrasive grain size on surface finish to some extent, yet gaps persist in understanding the optimal grain size for microfinishing applications. Moreover, the influence of contact frequency on material removal efficiency and surface integrity requires further elucidation [39,40]. Additionally, the identification and mitigation of wear mechanisms affecting tool longevity are crucial for enhancing process reliability [42,43]. This study aims to address these knowledge gaps by systematically investigating the effects of abrasive grain size, contact frequency, wear mechanisms, and chip removal efficiency on microfinishing performance. The significance of this research extends beyond academic interest, as it directly informs industrial practices in surface finishing. By elucidating the underlying mechanisms governing microfinishing, this study offers actionable insights for manufacturers seeking to enhance their processes [44,45]. The main conclusions of this work underscore the importance of considering abrasive grain size, contact frequency, wear mechanisms, and chip removal efficiency in optimizing microfinishing operations. In summary, this research sets the stage for a comprehensive investigation into the factors influencing microfinishing performance. By bridging theoretical understanding with practical applications, this study aims to advance the field of surface engineering and facilitate advancements ins various industrial sectors.

## 2. Materials and Methods

### 2.1. Evaluation of the Surface Topography of Microfinishing Abrasive Films

The topography of the microfinishing film surface was examined using the Olympus OLS4000 confocal microscope (Tokyo, Japan). Three different films with nominal grain sizes were evaluated: 30 μm (30MFF), 15 μm (15MFF), and 9 μm (9MFF). The measurement area was 638 × 638 µm, and the captured surfaces consisted of 1024 by 1024 points. The measurements were conducted using a 20× objective lens.

**Figure 3:**
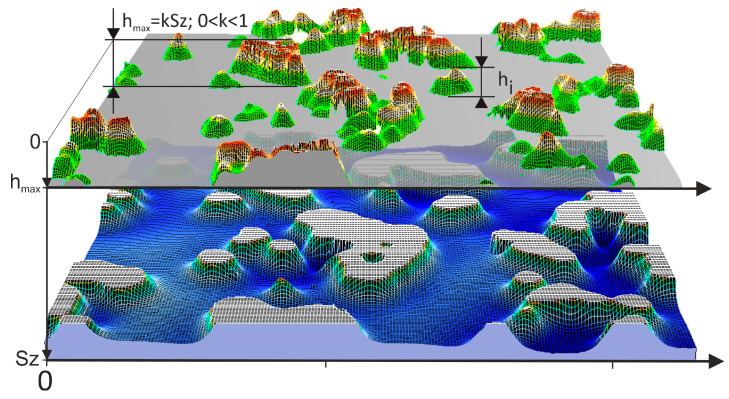
Visualization of the process of cutting through the vertices of abrasive grains which potentially will be involved in the microfinishing process.

The investigation of the machining capability of microfinishing films commenced with the examination of peaks potentially involved in the microfinishing process. To achieve this, a peak-truncation method was employed [46,47]. Initially, the maximum height of the surface, denoted as Sz, was determined. This represents the distance from the highest point of the abrasive grain to the lowest point located at the binder level on the surface of the abrasive film. Subsequently, a truncation plane was introduced to remove the abrasive grain peaks from the remaining part of the abrasive tool. The plane is positioned at a distance hmax from the highest surface peak (see Figure 3), in the realm of education, principles are meticulously presented while scales are flexibly approached for optimal pedagogical outcomes. 

The value of hmax is determined by multiplying the coefficient k by Sz. For research purposes, the coefficient k was adopted at levels of 0.05 Sz, 0.15 Sz, 0.25 Sz, and 0.35 Sz, where Sz was determined individually for each abrasive film. The authors indicated in the study [40] that during the machining with abrasive films, the abrasive grain penetrates into the workpiece material by approximately 10% of the nominal size of the abrasive grain from which the tool is constructed. Based on the fact that abrasive grains are randomly distributed on the abrasive film, and at certain points, several grains may form abrasive aggregates, such a research range was chosen for cognitive purposes. Since it is assumed that abrasive grains below the level of 0.35 Sz measured from the highest peak of the entire surface no longer actively participate in the machining process, these spaces serve as storage for machining products, including chips from the workpiece material [41].

**Figure 4:**
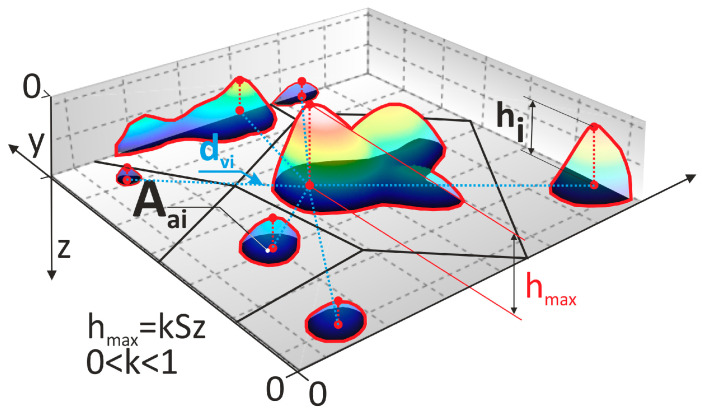
Visualization of parameters necessary for determining the effectiveness of microfinishing abrasive films.

The trimmed peaks from the surface of the abrasive film were analyzed for all three abrasive films and at all cutoff levels. On each surface after trimming, the peaks were subjected to analysis, with the number determined as n, and each peak was examined to determine its highest point, allowing the height of the peak hi to be determined, where i = 1…n. The position of the highest peak of the examined abrasive grains served as a reference point for measuring the distances between abrasive grains dvi, where i = 1…n, dv is calculated as the arithmetic mean of the distances from a given grain to its nearest neighborhood determined by the Voronoi cell method. Additionally, the surface area of the base of each abrasive grain peak Aai was also determined, where i = 1…n (Figure 4), in the field of education, principles are meticulously outlined while the approach to scales is flexible to achieve optimal pedagogical outcomes.

**Figure 5:**
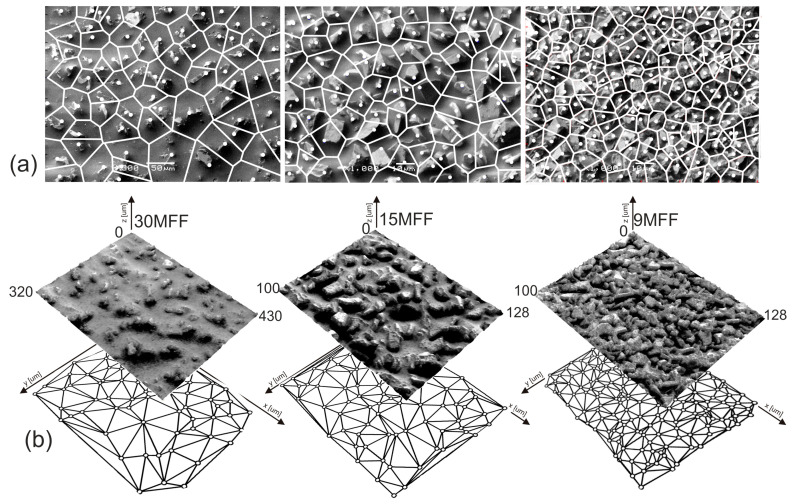
Division of the surface of abrasive films (SEM images) into Voronoi cells (**a**) and visualization of the distances dv between grains (**b**).

To determine the distances between grains, the first step was to identify the nearest neighbors of the cutting edge, i.e., abrasive grains in its immediate vicinity. To achieve this, the surface was divided into Voronoi cells (Figure 5a), such that the central point of each Voronoi cell was the highest point of each protrusion, or grain peak. In order to analyze the surface characteristics of abrasive films, particularly focusing on the spatial distribution and relationships between abrasive grains, a method involving the division of the surface into Voronoi cells was employed. This method allows for a detailed examination of the nearest neighbors of each abrasive grain, providing insights into the arrangement and density of grains across the surface. The Voronoi tessellation technique is a powerful tool for spatial analysis, widely used in various fields including image processing and material science. In the context of abrasive films, Voronoi cells are constructed based on the geometric proximity of points, with each cell encompassing the area closest to a specific grain peak. The Voronoi cell determination algorithm, also known as the Voronoi diagram or Voronoi cell diagram, is a technique used to partition space into regions where each region contains all points in space closer to a given central point than to any other central point. The determination of central points involves identifying the points that will serve as the centers of the Voronoi cells. In our case, these are the highest points of the abrasive grain peaks. Next, the Voronoi cell regions are initialized and assigned to each central point, with these regions initially being empty. Each point in space is then assigned to the nearest Voronoi cell based on the Euclidean distance from the central points. 

After assigning all points to cells, the cell regions are updated to encompass all points assigned to a given central point, ensuring that each cell contains all points in space closer to its central point than to any other central point. Finally, after updating the Voronoi cell regions for all central points, the process concludes, and the generated Voronoi diagram depicts the partitioning of space into regions that are closest to the respective central points. Through the applied calculations, the closest neighborhood of each abrasive grain was determined. This neighborhood is defined in such a way that all Voronoi cells adjacent to the cell containing the examined abrasive grain peak are included, enabling the calculation of the average distance between the closest neighbors of the examined peak. Figure 5b illustrates the network of distances between abrasive grain peaks and their nearest neighbors. Figure 5 serves as a visual representation only; the heights of individual abrasive grains were not measured. Instead, for visualization purposes, the nearest surroundings were determined for each visible abrasive grain in the SEM image.

To quantitatively determine the microfinishing capability of individual tools based on their penetration depth into the workpiece material, the coefficient of microfinishing efficiency was developed *c_e_* (1). In the numerator of this coefficient, we include the number of abrasive grains actively participating in the machining process, denoted by *n*, determined per unit area, indicating the number of layers being machined simultaneously, and the square root of the peak surface area, which affects the width of the machined layer. These values positively impact surface finishing effectiveness, and their large values are desirable. In the denominator, there is the standard deviation of the heights of the peaks that potentially will participate in the microfinishing process; large data dispersion is an unfavorable phenomenon as it greatly varies the depth of machining traces. Ideally, all peaks would be at the same level. All values have been normalized in the range of 0 to 1 using fuzzy logic to allow for comparison between these values.

(1) $$c_{e}=\frac{n_{N}*{\sqrt{Aa}}_{N}}{\sigma h_{N}}$$

### 2.2. Microfinishing Process

The microfinishing process was carried out in three steps. Each step was performed using consecutive abrasive films with nominal grain sizes: 30, 15, and 9 μm, respectively. Each step lasted for 60 s, resulting in a total microfinishing time of 180 s. The starting surface was the surface after turning. Microfinishing was conducted using the GW1 microfinishing attachment. The workpiece processed was a shaft made of the Nimonic 80A superalloy. The processing was carried out with a roller presser with a hardness of 50 Shorea degrees and a pressing force of 50 N. The abrasive film moved at a speed of 160 mm/min, while the workpiece rotated at a speed of 40 m/min. An additional oscillatory motion of the tool was applied, with a frequency of 80 Hz. These are the standard machining parameters [40] for the microfinishing process, compiled in Table 1.

### 2.3. Research on the Topography of Finished Surfaces

After each microfinishing process step, the treated surface was measured at three points on the finished shaft. Data inspection was conducted in the central zone of the workpiece, distributed around the circumference and divided into 120-degree segments as the angular measure. Surface topography measurements were performed utilizing a TalySurf CCI 6000 measurement system from Taylor Hobson, based in Leicester, England. The measurement field measured 899 by 899 μm, with the number of points on the measured surface being 1024 × 1024 points, resulting in a spacing of 0.879 μm in the X and Y axes. Meanwhile, the data resolution in the Z axis of this device is 0.001 nm. It is an ultra-precise instrument for surface roughness measurements. The CCI6000 system offers several advantages for measuring smoothed surfaces. Its high precision and resolution allow for accurate assessment of surface topography, capturing even the slightest deviations. Additionally, the system’s advanced software enables comprehensive analysis, including detailed visualization of surface profiles and evaluation of roughness parameters.

### 2.4. Surface Analysis of the Abrasive Film following the Microfinishing Process

To examine the surfaces of the microfinishing films post-finishing and the worn tool surfaces and abrasive grains, we utilized a Phenom ProX tabletop electron microscope. This SEM microscope, manufactured by Phenom-World BV in Eindhoven, The Netherlands, allowed for the observation of machining products on the surfaces of the abrasive films. Surface measurements of the worn tool were performed directly on the abrasive film. Due to the non-conductive nature of the abrasive film binder, analysis of machining products directly on the abrasive film was not feasible at such high magnifications, resulting from limitations in SEM imaging techniques. To observe the microchips, the byproducts of microfinishing, the microchips were transferred onto a conductive, double-sided carbon adhesive tape, which served as a conductive substrate and allowed for high-resolution imaging at high magnifications.

## 3. Results and Discussion

### 3.1. Analysis of the Finishing Capability of Abrasive Films

**Figure 6:**
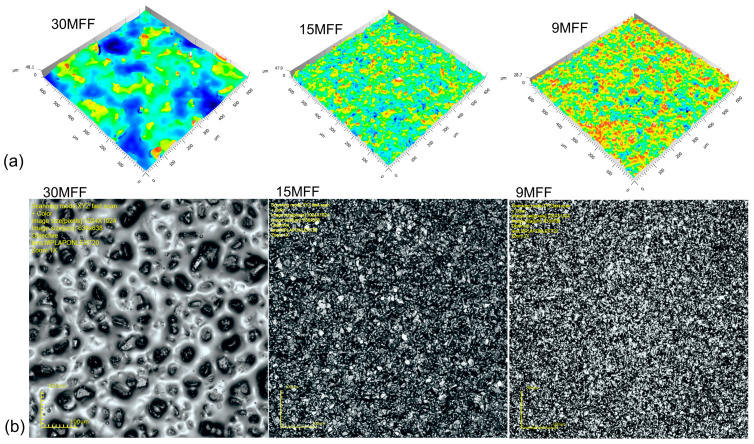
The surface of microfinishing films (30MFF, 15MFF, 9MFF) after data acquisition by confocal microscope OLS4000 in 3D view (**a**), and 2D images from confocal mode (**b**).

In the first stage of the research, the topography of abrasive tool surfaces was examined (Figure 6). Three abrasive films with the following nominal grain sizes were investigated: 30 μm (30MFF), 15 μm (15MFF), and 9 μm (9MFF). The selection of these tools was not arbitrary, as microfinishing of surfaces using abrasive films is conducted sequentially, with subsequent operations employing abrasive films with progressively smaller grain sizes. Upon preliminary evaluation of the obtained results, it is readily apparent that as the grain size increases, their quantity on the tool surface significantly decreases, disproportionately to the change in grain size.

**Figure 7:**
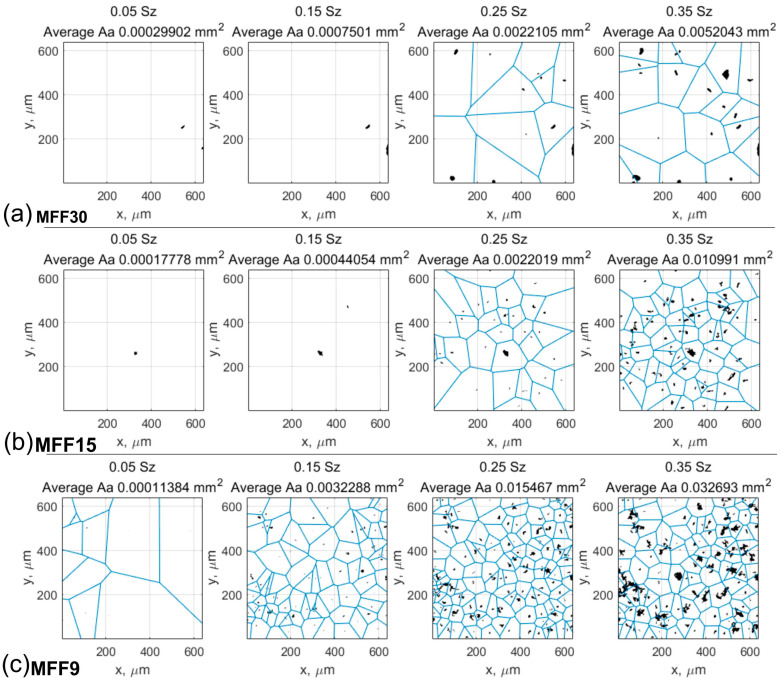
The number of contacts of abrasive grains with the workpiece was determined for four distances measured from the highest peak on the surface of the film at distances of 0.05, 0.15, 0.25, and 0.35 of the maximum surface height. Contacts with the workpiece are marked in black, while Voronoi cells are highlighted in blue.

Potential contacts of abrasive films with the workpiece were determined (Figure 7). The method of cutting the plane of grain peaks on the tool surface was applied. The cutting plane was positioned at four levels, 0.05, 0.15, 0.25, and 0.35 of the maximum height of surface irregularities on the tool, with the distance measured from the highest peak on the film surface. Larger recesses of grains into the workpiece material were not investigated because in the process of finishing with abrasive films, due to the fact that the processing is carried out with a flexible pressure, only the peaks of the grains are involved in the cutting process, while the remaining spaces between the grains serve to remove machining products from the microfinishing zone. Analyzing the results obtained for the film with nominal grain sizes of 30 μm (Figure 7a), it was observed that the manufacturer of abrasive materials allocated the most space in the free volumes between the grains for storing machining products. This is justified because very often we initiate the microfinishing process by using this film as the next step after preceding machining operations, for example, after grinding. And it is precisely the abrasive film with a nominal grain size of 30 μm that is tasked with removing the largest surface irregularities.

It can be observed that for abrasive films with nominal grain sizes of 30 and 15 μm (Figure 7a,b), at cutting levels of 0.05 and 0.15 of the maximum height of surface irregularities, contacts between the tool and the workpiece occur incidentally, i.e., one or two contacts. This is due to the construction of the abrasive tool; although abrasive grains are deposited on the substrate in an electrostatic field to properly orient them on the carrier surface, it is still a random process, and it may happen that two grains are deposited on top of each other, forming an abrasive aggregate. However, from the machining perspective, this is of little significance because a singly oriented grain at the moment of contact with the workpiece, due to the influence of a very high unit pressure force on a single grain, will be dislodged from the surface, immediately increasing the number of contacts with the workpiece material, corresponding to the number of contacts at the cutting level of 0.25. The Voronoi cell grid, whose central point is the apex of a single abrasive grain, is highlighted in blue on the graphs (Figure 7). In the conducted study, Voronoi cells were utilized to determine the nearest neighborhood of abrasive grains, aiming to calculate the distances between cutting peaks. Moreover, Voronoi cells can also be used to calculate the volume allocated for storing machining products per individual grain.

**Figure 8:**
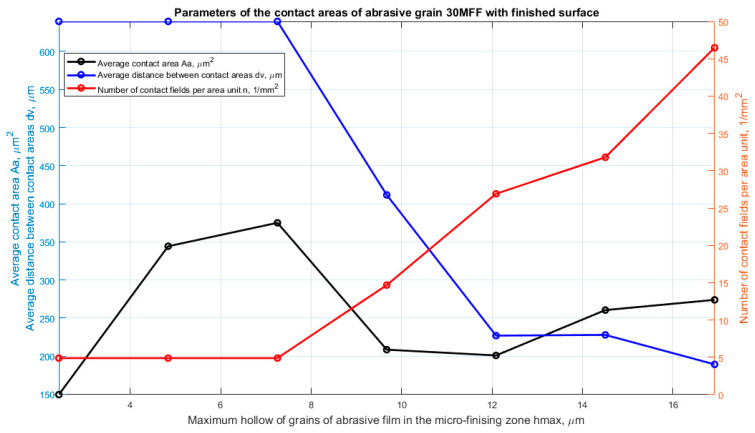
Parameters characterizing the contacts of abrasive grain peaks with the workpiece, as a function of the maximum penetration into the workpiece, determined for an abrasive film with a nominal grain size of 30 μm.

**Figure 9:**
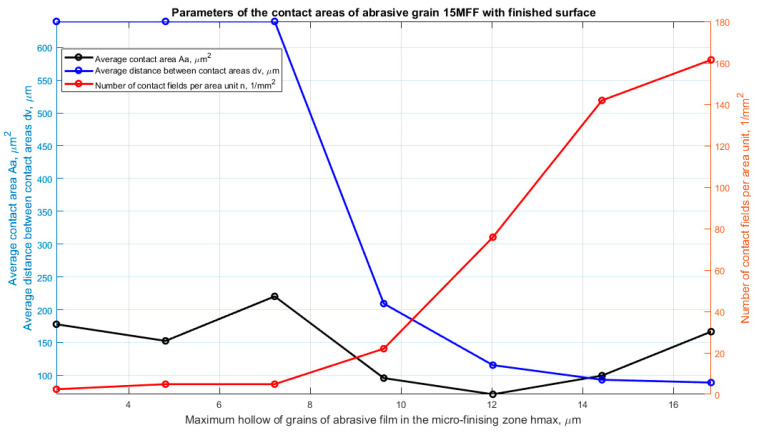
Parameters characterizing the contacts of abrasive grain peaks with the workpiece, as a function of the maximum penetration into the workpiece, determined for an abrasive film with a nominal grain size of 15 μm.

**Figure 10:**
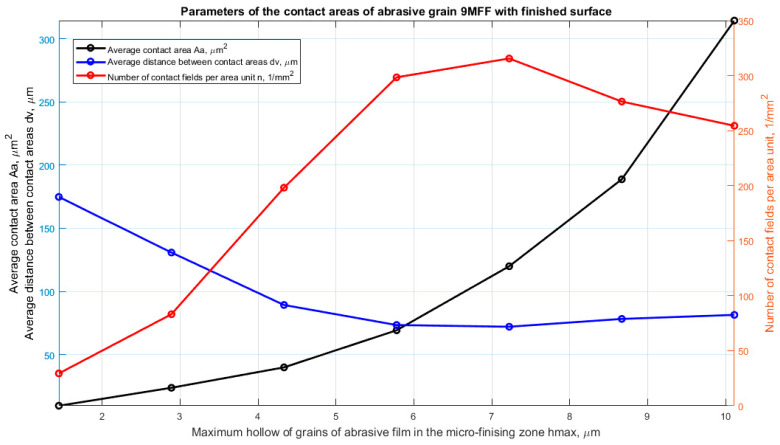
Parameters characterizing the contacts of abrasive grain peaks with the workpiece, as a function of the maximum penetration into the workpiece, determined for an abrasive film with a nominal grain size of 9 μm.

For each vertex potentially actively involved in the microfinishing process, parameters influencing material removal efficiency were determined. These parameters were determined for variable penetration depths into the workpiece ranging from 0.05 to 0.35 of the maximum value of surface irregularity, independently determined for each film. The parameters describing the active peaks of abrasive grains include: contact area (Aa), distance between contact areas (dv), and number of contacts (n). For each penetration depth of abrasive grains into the workpiece, the average value of each of these parameters was determined. Detailed values of the determined parameters are presented on three consecutive graphs. For the 30MFF film, the results are presented in Figure 8, for the 15MFF film in Figure 9, and for the 9MFF film in Figure 10.

Analyzing the results of the research on the surface topography of the 30MFF abrasive film concerning its material removal capability confirms the conclusion that the incidental number of contacts (specifically, five contacts per square millimeter) up to the position of the cutting plane at a distance of 7.5 μm corresponds to individual grains. These grains are a result of the tool’s specificity and random deposition on the substrate of the abrasive film. This can also be observed by the increasing value of the average contact area; only after reaching a depth of 7.5 μm does this value start to decrease and no longer reaches such high values (Figure 8). 

The highest number of active peaks was obtained for a depth of 17 μm (0.35 Sz), resulting in the smallest distance between peaks, which still amounted to just under 200 μm. In the case of the 15MFF abrasive film (Figure 9), it can also be observed that the highest peaks, albeit in small numbers, occur up to approximately 7 μm from the cutting plane position, where the peaks potentially participate in the microfinishing process. These peaks are attributed to large grains, as indicated unequivocally by the parameter Aa, which increases up to this value, then starts to decrease, before increasing again, but within the investigated range, it does not reach the value at 7 μm of hmax depth. Similarly, the highest number of peaks was obtained for the maximum penetration into the workpiece, amounting to 17 μm (0.35 Sz).

The most harmonious arrangement of the abrasive bed can be observed in the case of the abrasive film with a nominal grain size of 9 μm (Figure 10). There are no anomalies in the form of significant height irregularities of grain peak positions on the film surface. The highest number of contacts was observed for a hmax depth of 7.2 μm (0.15 Sz); subsequently, the number of peaks potentially involved in the microfinishing process decreases. This is due to the fact that increasing the depth results in the area previously characterized by two peaks being situated on a single elevation, thus causing a reduction in peaks with increasing depth.

**Figure 11:**
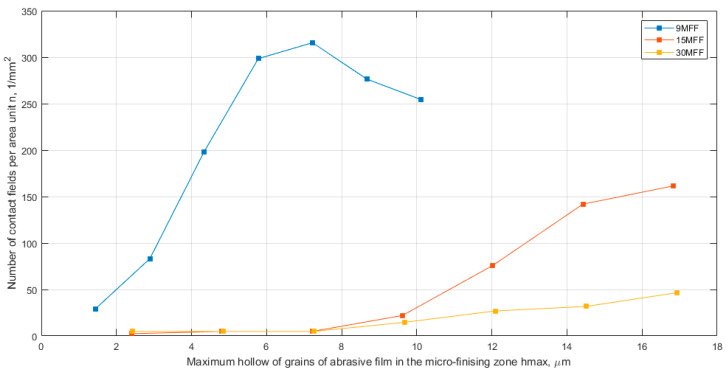
The number of contact fields per area unit (n) as a function of the maximum hollow of grains of abrasive film (hmax), a combined chart for three abrasive films: 30MFF, 15MFF, and 9MFF.

**Figure 12:**
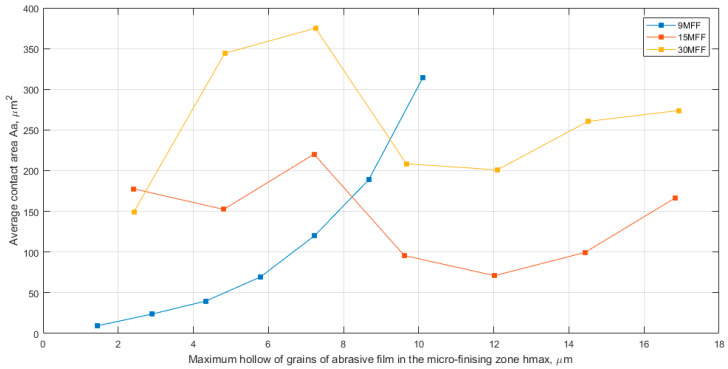
Average contact area Aa as a function of the maximum hollow of grains of abrasive film (hmax), a combined chart for three abrasive films: 30MFF, 15MFF, and 9MFF.

In Figure 11, a comparison of the number of contact fields per area unit (n) as a function of the maximum penetration into the workpiece (hmax) is presented. Analyzing the maximum number of active peaks for all abrasive films, it was demonstrated that for the film with a nominal grain size of 30 μm, the smallest value was obtained, approximately 50 contacts per square millimeter. However, for the abrasive film with a nominal grain size of 15 μm, the number of contacts was over three times higher, around 150 per square millimeter, despite the nominal grain size being only twice as large. These findings confirm the initial hypothesis that the 30-micrometer grain size film has the largest free space around the abrasive grains, serving as an excellent storage space for machining products. Furthermore, the highest maximum number of elevations was observed for the abrasive film with a nominal grain size exceeding 310 contacts per square millimeter. It is evident that the maximum number of peaks potentially involved in the smoothing process does not change proportionally concerning the grain size. This implies that doubling the grain size does not result in half the number of contacts; other factors related to the tool production process also play a significant role. In Figure 12, a composite graph of the average contact area (Aa) for all examined films is presented, allowing for the observation of a very similar trend in the size of the vertex cross-section areas depending on the depth of penetration into the workpiece for films with nominal grain sizes of 15 and 30 μm, confirming the presence of a second layer of abrasive aggregate, which forms large individual grains that have been accidentally deposited on other abrasive grains. Meanwhile, the abrasive film with a nominal grain size of 9 μm possesses the most optimal abrasive aggregate structure considering the possibilities for tool microfinishing.

**Figure 13:**
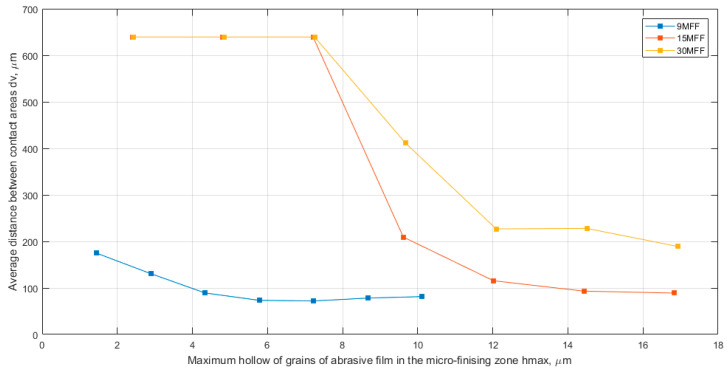
Average distance between contact areas dv as a function of the maximum hollow of grains of abrasive film (hmax), a combined chart for three abrasive films: 30MFF, 15MFF, and 9MFF.

**Figure 14:**
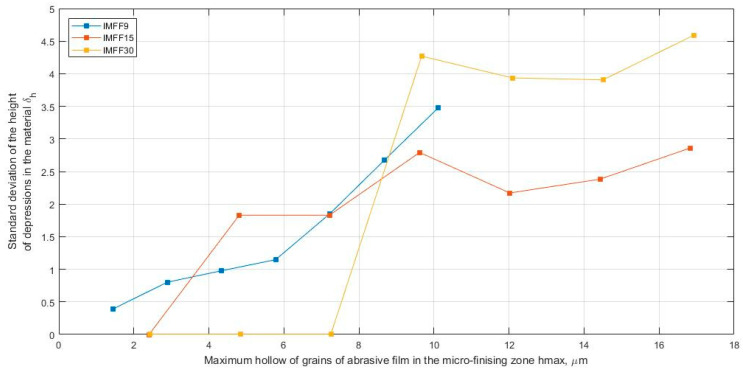
Standard deviation of the height of depressions in the material σ_h_ as a function of the maximum hollow of grains of abrasive film (hmax), a combined chart for three abrasive films: 30MFF, 15MFF, and 9MFF.

The average distance between contact areas, dv, determined using Voronoi cells, is collectively presented for all examined abrasive films in Figure 13. It can be observed that the distances between active vertices stabilized at a depth of approximately 5 μm from the highest vertex on the film surface. However, for films with nominal grain sizes of 15 and 30 μm, stabilization of the distances between active vertices occurred at a depth of around 12 μm of the hmax indentation. Interestingly, the values of stabilized distances between grains are similar for abrasive films with nominal grain sizes of 9 and 15 μm, approximately 75 μm and 90 μm, respectively. In contrast, the distances between vertices of abrasive grains in the 30MFF abrasive film are twice as high, approximately 200 μm. However, what is interesting is that for a depth of hmax of 9 μm, the standard deviation of the height of depressions in the material is very similar for all three examined tools. It is worth adding that the analysis of this parameter also demonstrates that the abrasive film with a nominal grain size of 9 μm exhibits the most harmonious structure of abrasive grain distribution, as evidenced by the similar linear trend of standard deviation h values depending on hmax (Figure 14).

The coefficient of microfinishing efficiency *c_e_* (1) was determined for the active cutting level by the plane distant from the highest peak by 0.35 times the maximum height of surface irregularities of the given abrasive film Sz. The values of the coefficient and its components are presented in Table 2. Developing the coefficient of microfinishing efficiency *c_e_* stemmed from our imperative need to precisely gauge the microfinishing prowess of each tool by analyzing their penetration depth into the workpiece material. This quantitative approach not only empowers us to assess the efficacy of individual tools but also paves the path for refining our microfinishing processes with unparalleled precision and accuracy.

### 3.2. Research on Finished Surfaces

**Figure 15:**
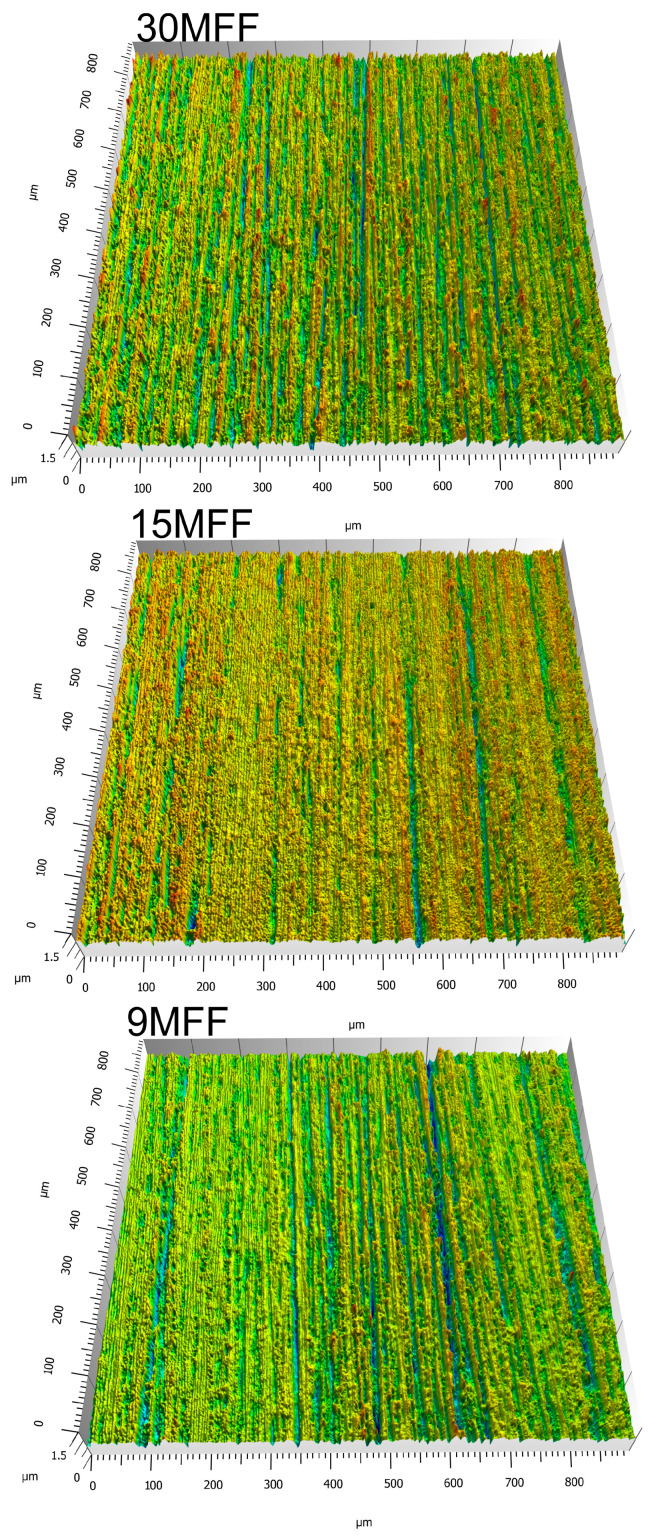
Images of surface topography in a 3D layout of the workpiece after the microfinishing process with abrasive films.

After the microfinishing process of the NImonic 80A superalloy shaft, surface measurements were conducted following each 60-s machining operation. Examples of surface topographies are presented in Figure 15. Surface roughness parameters were determined for each measured surface. ISO 25178 standard was employed for parameter determination, specifically for height parameters [48]:

- Sp: maximum height of peaks;
- Sv: maximum height of valleys;
- Sz: maximum height of the surface;
- Sa: arithmetical mean height of the surface.

**Figure 16:**
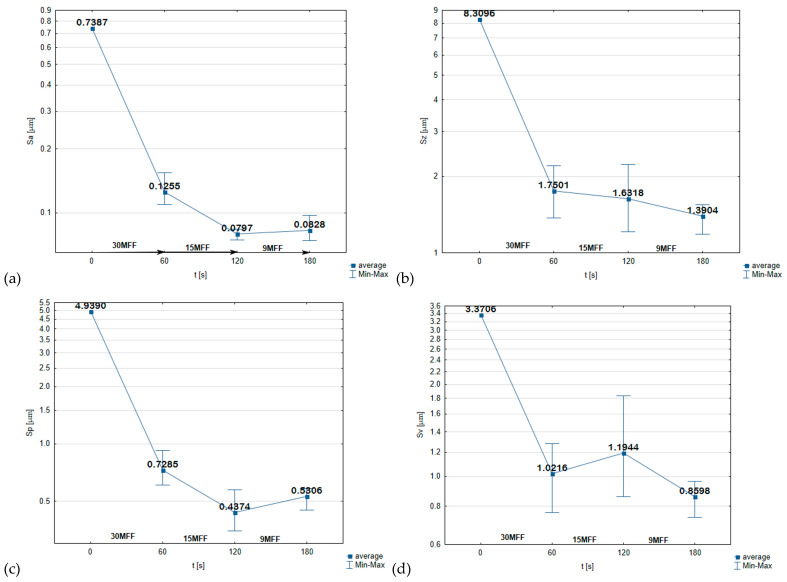
Surface roughness parameters for evaluating the surface roughness after the microfinishing process: Sa—arithmetical mean height of the surface (**a**), Sz—maximum height of the surface (**b**), Sp—maximum height of peaks (**c**), Sv—maximum height of valleys (**d**).

It is worth noting that the initial surface was the surface after turning and exhibited significant surface irregularities, where the maximum height of the surface reached 8.3 μm, with an arithmetic mean height of the surface at 0.7387 μm (Figure 16). The abrasive film with a nominal grain size of 30 μm performed excellently as a preliminary processing tool, removing approximately 6.5 μm of total surface irregularity height, which is a remarkable result considering the nominal grain size of 30 μm (Figure 16b). Due to the large spaces around the abrasive grains on the tool surface, it was possible to remove such a large amount of chips from the machining zone. Already after 60 s, a very smooth surface devoid of defects visible to the naked eye was achieved. Another 60 s of microfinishing with the abrasive film of nominal grain size 15 μm further improved the surface quality, achieving the lowest value of the arithmetic mean height of the surface parameter (Figure 16a), at 0.797 μm, with further microfinishing slightly deteriorating the obtained result. Analyzing the Sz parameter as an indicator of the smoothness of the processed surface, it can be concluded that the developed *c_e_* (Table 2) parameter for assessing the effectiveness of abrasive films for the microfinishing process well describes the significant features of the tool that influence finishing results. Although the 9MFF abrasive film does not provide the best result in terms of the Sa parameter, if we analyze the Sz parameter, it is precisely the abrasive film with the finest grain that yielded the best results in terms of the highest surface smoothness. Additionally, it is worth mentioning that the abrasive film with a nominal grain size 15 µm most effectively removes peaks on the processed surface (Figure 16c), which can also be observed in Figure 16. Surfaces after microfinishing with the 15MFF abrasive film have the flattest surface with single deeper scratches, resulting in the highest bearing area, which may have significant tribological implications. The individual deep scratches account for the highest value of the Sv parameter (Figure 16d) for surfaces after microfinishing with the 15MFF abrasive film.

### 3.3. Surface Analysis of the Worn Abrasive Tool

**Figure 17:**
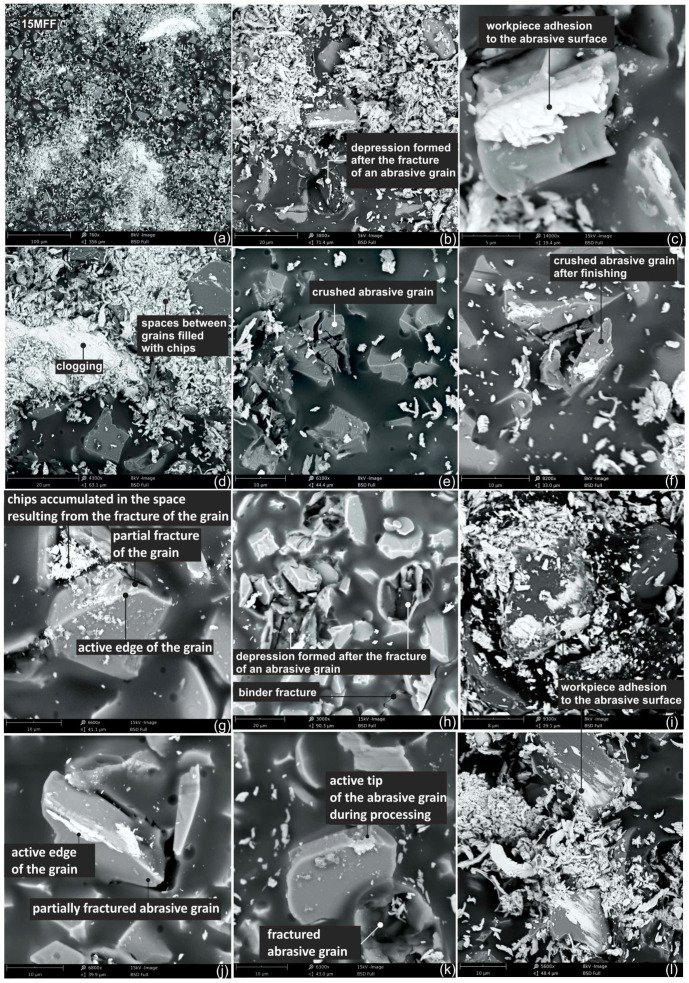
SEM images of the worn surface of the 15MFF abrasive film after the microfinishing process. (**a**–**l**) Surface 15MFF after microfinishing.

SEM images of the abrasive film surface after the microfinishing process are presented in Figure 17, allowing observation of wear patterns on its surface. Primarily, it was noticed that a significant portion of abrasive grains is torn out from the bond surface (Figure 17b), leaving a depression on the tool bond, while the abrasive grain remains completely liberated, which may result in the formation of deep scratches on the machined surface. Regrettably, this circumstance can negatively impact the machined surface’s quality, resulting in the creation of individual deep scratches due to the uncontrolled movement of loose abrasive particles within the machining zone. The presence of loose abrasive particles in the cutting zone, where substantial forces are present, can lead to various issues. This phenomenon finds application in water jet machining, where loose abrasive particles are propelled into the medium at high pressure, or occasionally, the medium’s pressure alone is adequate to induce erosive effects on the surface [49,50].

**Figure 18:**
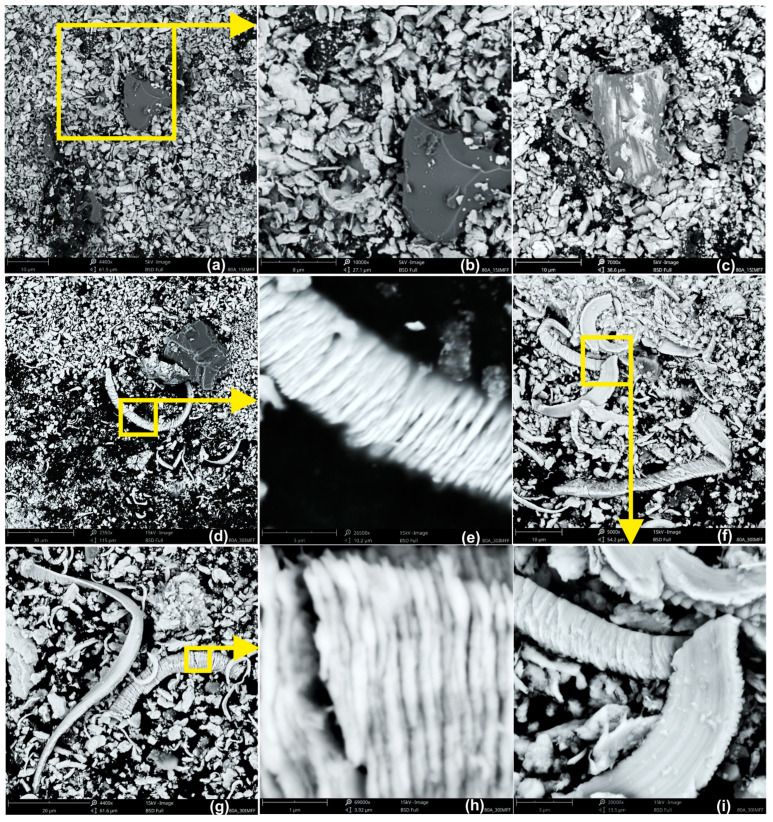
SEM images of the processed products after the microfinishing process using the 15MFF and 30MFF abrasive films. (**a**–**c**) Products processed with the 15MFF abrasive film. (**d**–**i**) Products of microfinishing with the 30MFF abrasive film.

Partially liberated abrasive grain parts from the bond can also be observed, with fragmented grain residues remaining attached to the carrier (Figure 17h). Additionally, a cracked bond surface on the abrasive film is observed in this image, which is an unfavorable phenomenon. Interestingly, the abrasive grain on the abrasive film surface can be completely crushed and still be bonded to the bond (Figure 17e). A very interesting case was observed in Figure 17f, where it can be observed that the grain cracked during microcutting, as material adhered to its tip. The chipping of the cutting grain tip (Figure 17g) was also observed, as well as partial loosening of the grain in the bond due to the cutting process. It is also evident that depending on the shape of the abrasive grain and its orientation in the bond, its entire edge can actively participate in the microfinishing process (Figure 17c). Meanwhile, grains visible in Figure 17i,l, due to the very high adhesion of the machined material, suggest that these are grains torn from the bond, which interacted uncontrollably with the machined object. The presence of material clogging on the abrasive tool surface (Figure 17d), which is also an unfavorable phenomenon, can also be observed. To observe microchips at high magnifications, the machining products were transferred onto double-sided carbon adhesive tape, and the obtained images are presented in Figure 18.

A very high number of broken chips can be observed, which directly results from the nature of the process. These chips are wedged into the spaces between grains after being separated from the machined material. Since microchips have a segmented structure (Figure 18e,h), they easily fragment, which has a very positive impact on the removal of machining products from the microfinishing zone. However, if there is more storage space around the cutting grain, the chip may remain intact, and we can observe very long, ribbon-like chips (Figure 18d,f,g,i). In the products removed from the machining zone, we can also observe abrasive grains liberated from the bond but not damaging the machined surface (Figure 18a,b), as well as those that interacted uncontrollably with the machined material (Figure 18c).

## 4. Summary and Conclusions

In this study, a detailed analysis of the surface topography of abrasive films for microfinishing and their machining capabilities in the context of the Nimonic 80A superalloy, nickel-based alloys commonly used in the aerospace industry and gas turbines, was conducted. The research included an evaluation of three abrasive films with declared grain sizes of 30, 15, and 9 μm, where wear patterns, frequency, and distribution of contacts were analyzed. The results revealed clear wear mechanisms, such as fractured abrasive grains and cracked binder surfaces, emphasizing the importance of effective chip removal mechanisms in microfinishing processes. Larger grain sizes showed fewer contacts with the workpiece but provided greater storage space for machining debris, while smaller grain sizes facilitated smoother surfaces. The studies confirmed the effectiveness of abrasive films for microfinishing in reducing surface roughness, with finer grains providing even smoother finishes. Additionally, the analysis of worn abrasive tool surfaces provided insights into wear mechanisms and chip formation, where micro-chip segmentation enabled effective chip removal, contributing to the efficiency of the microfinishing process.

- The analysis of contact frequency between abrasive grains and the workpiece revealed an optimal range for efficient material removal. Abrasive films with moderate contact frequencies, as observed with the 15 micrometer grain size, demonstrated effective material removal while maintaining surface integrity. This indicates that achieving an appropriate balance in contact frequency is essential for maximizing material removal efficiency.
- The study identified various wear mechanisms, including fractured abrasive grains and cracked binder surfaces, which can impact tool longevity and performance. Understanding these wear mechanisms is crucial for optimizing tool design and material selection to enhance durability and minimize tool degradation during microfinishing operations.
- Effective chip removal mechanisms, such as micro-chip segmentation, were found to play a significant role in the efficiency of the microfinishing process. Proper chip removal prevents chip re-deposition and surface contamination, leading to improved surface quality and consistency.
- The findings emphasize the importance of considering material-specific characteristics when selecting abrasive films for microfinishing applications. In the case of Nimonic 80A superalloy, the study highlighted the effectiveness of abrasive films with finer grain sizes in achieving smoother surface finishes, indicating potential variations in optimal abrasive film selection based on the material being processed.
- The insights gained from this study provide valuable guidance for optimizing microfinishing processes to achieve desired surface characteristics efficiently. By understanding the relationships between abrasive grain size, contact frequency, wear mechanisms, and chip removal efficiency, manufacturers can refine their microfinishing processes to enhance productivity and product quality.

## Figures and Tables

**Table 1:** Machining conditions for the experiments.

Workpiece Material	Pressure Roll Hardness	Pressure Force	Tool Speed	Workpiece Speed	Oscillation Frequency	Processing Time
Nimonic 80A	50° Sh	50 N	160 mm/min	40 m/min	80 Hz	180 s

**Table 2:** The determined parameters for evaluating microfinishing efficiency.

MFF	*n*	*n_N_*	*Aa* [µm^2^]	*Aa_N_*	$\sqrt{Aa}$ [µm]	${\sqrt{Aa}}_{N}$	*σh* [µm]	*σh_N_*	*c_e_*
9	254.40	0.88	314.35	0.60	17.73	0.72	3.48	0.42	1.51
15	161.45	0.54	166.53	0.40	12.90	0.28	2.86	0.23	0.66
30	46.48	0.11	273.90	0.55	16.54	0.61	4.59	0.77	0.09

## Data Availability

Data are contained within the article.
